# For Vomiting from Anaesthetics

**Published:** 1898-02

**Authors:** 


**For Vomiting from Anaesthetics.**—The following is often useful for the relief of obstinate vomiting which follows an anæsthetic:

℞ Rectified spirit...........................10 parts

Menthol.................................. 4 "

Tincture of nux vomica............. 2 "

M. Sig.: Ten drops to be taken every hour in a teaspoonful of cherry laurel water.—*American Journal of Surgery and Gynecology.*

